# Towards a More Precise Serological Diagnosis of Human Tegumentary Leishmaniasis Using *Leishmania* Recombinant Proteins

**DOI:** 10.1371/journal.pone.0066110

**Published:** 2013-06-12

**Authors:** Ana Paula Souza, Manuel Soto, Jackson M. L. Costa, Viviane S. Boaventura, Camila I. de Oliveira, Juqueline R. Cristal, Manoel Barral-Netto, Aldina Barral

**Affiliations:** 1 Centro de Pesquisas Gonçalo Moniz (CPqGM), Fundação Oswaldo Cruz (FIOCRUZ), Salvador, Bahia, Brazil; 2 Centro de Biología Molecular Severo Ochoa (CSIC-UAM), Departamento de Biología Molecular, Universidad Autónoma de Madrid, Madrid, Spain; 3 Faculdade de Medicina da Bahia, Universidade Federal da Bahia (UFBA), Salvador, Bahia, Brazil; 4 Instituto Nacional de Ciência e Tecnologia de Investigação em Imunologia (iii-INCT), Salvador, Bahia, Brazil; University of São Paulo, Brazil

## Abstract

**Background:**

Exposure to *Leishmania* induces a humoral immune response that can be used as a marker of parasite exposure.

**Methodology/Principal Findings:**

Herein, ELISA was used to screen sera from patients with Tegumentary Leishmaniasis (TL) against different *L.* infantum-*chagasi*-derived recombinant proteins (rHSP70, rH2A, rH2B, rH3, rH4 and rKMP11). Among the recombinant proteins, rHSP70 and rH2A showed the best reactivity against human sera obtained from endemic areas of TL. Receiver-Operator Characteristics (ROC) curve analysis was used to identify the effectiveness of these proteins for serodiagnosis of TL. ROC curves confirmed the superior performance of rHSP70 and rH2A, in comparison to the other tested recombinant proteins. Additionally, we evaluated the specificity of the response to rHSP70 and rH2A by testing sera obtained from patients with Chagas' disease, Tuberculosis, Leprosy or Systemic Lupus Erythematosus. In this case, rHSP70 displayed an increased ability to discriminate diseases, in comparison to SLA.

**Conclusion:**

Our results raise possibility of using rHSP70 for the serodiagnosis of TL

## Introduction

The success of Leishmaniasis treatment depends on an effective and early diagnosis. Currently, diagnosis of Tegumentary Leishmaniasis (TL) is often performed based on clinical and epidemiological data associated with the results of different laboratory tests. Parasitological diagnosis is highly specific as it demonstrates the presence of the parasite by immuno-staining of biopsy tissues, by PCR amplification of parasite DNA or, ultimately, by cultivation of biopsy material allowing for parasite growth or for experimental inoculation into susceptible laboratory animals [1]–[11]. These methods have high specificity, but sensitivity is subject to variation because the tissue distribution of parasites is not homogeneous [6]–[8]. Moreover, parasitological tests require invasive procedures and depend on restrictive conditions for the collection of material which limit their use in large-scale epidemiological studies. Due to these reasons, indirect tests, based on the host immune response are of utmost importance.

Sera from Leishmaniasis patients are routinely collected for laboratory evaluation of several parameters and serological tests for infectious diseases are easy to perform. Despite new approaches for immunodiagnosis [12], the Enzyme-Linked Immunosorbent Assay (ELISA) has been the most widely used serological method for Leishmaniasis diagnosis as it is easy to perform and has a low cost (reviewed in [13]). In addition, this method can be performed in intermediate level laboratories using relatively simple equipment [13]. ELISA is commonly used to diagnose visceral leishmaniasis (VL) and has a higher sensitivity for TL diagnosis than parasitological tests (reviewed by [6] and [14]).

Crude *Leishmania* antigen preparations, also known as soluble *Leishmania* antigen (SLA), are the most commonly parasite proteins employed in ELISA for Leishmaniasis diagnosis. Although the techniques performed with SLA have high sensitivity, they lack specificity. Use of SLA and occurrence of false positive results in cases of Chagas disease has been documented [15]–[19]. Additionally, there is an important variation in SLA preparations reflecting on ELISA sensitivity [14]. Aiming to increase ELISA specificity in the diagnosis of VL or TL, through the elimination of cross-reactive epitopes, the use of recombinant *Leishmania* inmunodominant antigens has been evaluated [20], [21]. Many recombinant antigens have been tested for the serodiagnosis of canine or human VL [22]–[24], however, up to date, few recombinant proteins have been described as promising for the serodiagnosis of human TL [25]. In the present study, we employed a large panel of human sera to test a series of parasite-derived recombinant proteins, using ELISA, aiming at the development of better tool for human TL serodiagnosis.

## Materials and Methods

### Soluble *Leishmania* Antigen

SLA was prepared from *L. infantum-chagasi* (MCAN/BR/00/BA262) promastigotes maintained in Schneider's medium (Sigma) supplemented with 10% inactivated fetal bovine serum, 100 U/ml penicillin and 100 ug/ml streptomycin (Gibco). The parasites were initially submitted to 10 alternating cycles of freezing and thawing in liquid nitrogen and water bath and then centrifuged at 1600× g, 4°C for 15 min. The supernatant containing SLA was collected and protein content quantified using the Micro BCA TM Protein Reagent Kit assay (Pierce).

### *Leishmania* recombinant proteins

*L. infantum-chagasi* derived histones [26] and KMP-11 [27] were expressed and purified as described. *L. infantum-chagasi* HSP70 was expressed after BamHI-HindIII cloning of the DNA insert containing the HSP70 coding region (Acc. Number. X85798) into the pQE30 prokaryotic expression vector. The DNA insert was obtained by PCR amplification using oligonucleotides: forward 5′- GC*GGATCC*ATGACATTCGAAGGCGCCAT -3′ and reverse 5′-GG*AAGCTT*TTAGTCGACCTCCTCGACCTTGG-3′ (in italics the additional sequences included for cloning purposes) and *L. infantum* (MCAN/ES/96/BCN150) DNA as template. Recombinant HSP70 was expressed and purified as described elsewhere [26].

### Sera

Samples for the present study were randomly selected from a serum bank (LIP-CPqGM-FIOCRUZ) built following from independent studies previously conducted in Northeastern Brazil, in areas endemic for cutaneous and mucosal Leishmaniasis [28], [29]. The sera panel consisted of 49 sera from Cutaneous Leishmaniasis (CL) and of 53 sera from Mucosal Leishmaniasis (ML) patients.

All TL patients had the diagnosis of Leishmaniasis confirmed by a positive result in at least two of the following tests: Montenegro skin test, anti-Leishmania serology, histopathology of the lesion or a therapeutic test. All TL patients came from areas endemic for TL and presented lesions with clinical characteristics compatible with either TL or CL what increases the positive predictive values of immunological and therapeutical tests.

### Ethics Statement

All samples used were anonymized. The study was approved by the Institutional review Board at Centro de Pesquisas Gonçalo Moniz.

### Study procedure

To evaluate sensitivity, ELISA was performed as described below with recombinant proteins (HSP70, H2A, H2B, H3, H4 and KMP11) and results were compared with SLA using the panel of sera from TL patients The ELISA cut-off value for each product was established from ROC curves, and calculated by comparison of the reactivity values from patient's serum samples and from normal volunteers (NV) from endemic (n = 39) and non-endemic (n = 49) areas. Recombinant proteins (HSP70, H2A, H2B, H3, H4 and KMP11) were then tested against a panel of sera from patients with Chagas disease (n = 30), Systemic Lupus Erythematosus (SLE) (n = 10), Leprosy (n = 30) and Tuberculosis (n = 22). The resulting OD values were compared with those obtained with the panel of TL sera. The epidemiological characteristics of individuals involved in the study are presented in Table 1.

**Table 1:** Epidemiological characteristics of individuals involved in the study.

Group	Age [median (extremes)]	Male [n (%)]	Female [n (%)]
CL	29.6 (8–72)	30 (61.2)	19 (38.8)
ML	37.8 (12–75)	35 (66)	18 (34.0)
Endemic controls	35.5 (15–65)	20 (51)	19 (49.0)
Non-endemic ctls	33.6 (20–58)	28 (57)	21 (43.0)
Chagas' disease	39.9 (22–56)	16 (53.3)	14 (46.7)
LES	34.5 (22–66)	0	10 (100)
Leprosy	37.7 (15–75)	13 (43.4)	17(15–75)
Tuberculosis	39.5 (18–85)	14 (63.6)	8 (36.4)

CL = Cutaneous Leishmaniasis; ML = Mucosal Leishmaniasis; Non-endemic ctls = Non-endemic controls; LES = Lupus Erythematosus Systemic.

### ELISA to detect anti-*Leishmania* antibodies

Anti-*Leishmania* serology was performed by ELISA as described by [30] with some adaptations. For this, 96-well plates were coated with SLA (10 ug/mL) or with *Leishmania* recombinant proteins (rH2A, rH2B, rH3, rH4, rHSP70 or rKMP11) (1 ug/ml) in carbonate buffer (0.45 M NaHCO_3_, Na_2_CO_3_, 0.02 M pH 9.6) for 12 h at 4°C. After three washes with PBS-0.5% Tween, the plates were blocked for 1 hour at 37°C with PBS Tween 0.5% plus 5% skim milk. Sera were diluted 1∶100 in PBS-Tween 0.5% plus 5% skim milk and incubated for 2 hours at room temperature, under agitation. After a final round of washing, the wells were incubated with anti-human IgG conjugated to alkaline phosphatase (Sigma, Louis, MO) at a 1∶2500 dilution in PBS-Tween 0.5% plus 5% skim milk for 1 hour at room temperature under agitation. Again the plates were washed and incubated for 30 minutes with a chromogenic solution of p-nitrophenyl phosphate in sodium carbonate buffer pH 9.6 with 1 mg/mL of MgCl_2_. Concentration of SLA and of recombinant proteins was determined in a dose-response experiment to evaluate an optimal signal without loss of specificity (data not shown). In all experiments, the values obtained were subtracted from those obtained in the background. The serological experiments were repeated twice yielding similar results. The tests with the recombinant proteins were conducted blind with regards to the results obtained with ELISA using SLA.

### Statistical analysis

Statistical analysis was performed using Prism 5.0 (GraphPad Prism Inc., San Diego, CA). Differences in values of antibody levels between groups were compared using the Kruskal-Wallis test with Dunn post-test. ROC curves (Receiver Operating Characteristic) were calculated for each recombinant protein. The cut-off, as determined by high sensitivity and specificity, from the highest probability of discrimination established by the curve, was therefore determined. The performance of each *Leishmania* recombinant proteins was established by the parameters obtained from ROC curves values with AUC, p values and likelihood ratio.

## Results

**Figure 1:**
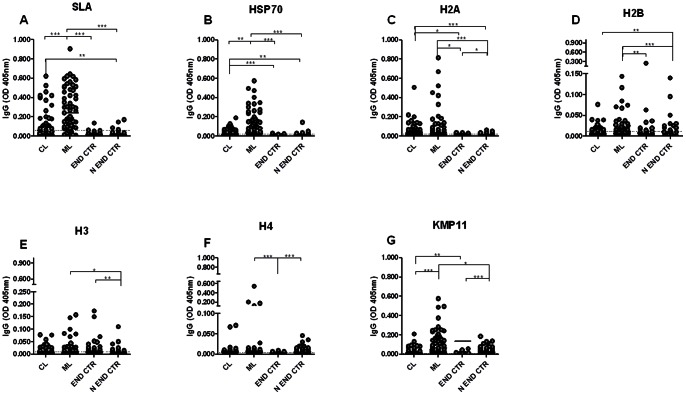
Anti-*Leishmania* IgG antibodies levels TL patients. Sera from mucosal *Leishmania*sis (ML) (n = 53) and cutaneous *Leishmania*sis (CL) (n = 49) patients, and sera from healthy individuals from endemic (n = 39) and from non-endemic (n = 49) areas were tested against SLA (A), rHSP70 (B), rH2A (C), rH2B (D), rH3 (E) rH4 (F) and rKMP11 antigens (G). The cut-off was calculated by comparison of the reactivity values from patient's serum samples and from normal volunteers from endemic area and non-endemic areas. The cut-off value for negative and positive samples is indicated in dotted line. The solid lines represent the median values. Each point represents the mean of the duplicate OD values for the same serum with a standard deviation lower than 20%. Significance was compared using Kruskal-Wallis test with Dunn's post test for multiple comparisons. * p<0.05, ** p<0.001 **, p<0.0001.

Initially we have evaluated the reactivity of the recombinant *Leishmania* proteins against a panel of sera from CL and ML patients. Overall, sera from ML patients presented a higher reactivity to the majority of the antigens tested, especially SLA (Fig. 1A) and rHSP70 (Fig. 1B), compared to CL patients. Regarding control sera (endemic and non endemic), a few false positive results were observed both with SLA (Fig. 1A), rHSP70 and rH2A (Fig. 1A–C). The other recombinant proteins show higher non-specific reaction against natural antibodies of healthy individuals resulting in a higher number of false positive samples (Fig. 1D–G). Regarding sera from TL patients (CL and ML), SLA gave a positive result with 46.9% of CL sera and with 85% of ML sera (Fig. 1A). Recombinant HSP70 yielded a positive response in 51.9% of CL and in 80% of ML sera (Fig. 1B). Among the histones, rH2A showed the best results with recognition of 72.2% of CL and of 56.4% of ML patients' sera (Fig. 1C). The remaining recombinant histones, in addition to having a significant number of false positive samples, were less recognized by sera from CL and from ML patients (Fig. 1D–F). Despite the recognition of rKMP11 by a significant percentage of sera from CL (55.5%) and ML (85.1%) patients, this antigen presented a high recognition (85.7%) by endemic control sera (Fig. 1G).

**Figure 2:**
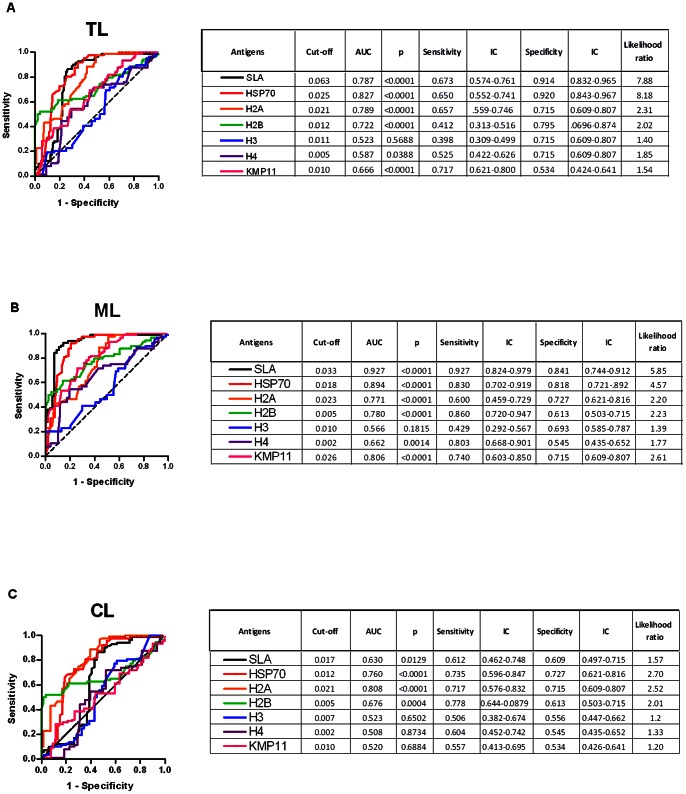
ROC curve of antibody levels predict the positivity thresholds against parasite antigens. The ROC curves were constructed using data obtained in ELISA performed with antigens and sera from TL (n = 102) (A), ML (n = 53) (B) or CL (n = 49) (C) patients and control subjects from an endemic (n = 39) and from a non-endemic (n = 49) area. The solid lines represent the area under the curve and the dotted line represents the identity line curves. The tables show the detailed information obtained for each ROC curve (cut-off values chosen, area under the curve, the values of p, and the sensitivity and specificity with a confidence interval of 95% and the likelihood ratio).

Based on the ELISA results (Fig. 1), ROC curves were calculated to evaluate the capacity of SLA and of each recombinant protein to discriminate TL patients from healthy volunteers (endemic and non endemic area). Comparisons were made considering all TL patients as well ML and CL patients, separately. As shown in Fig. 2A, for TL patients (including ML and CL patients) the best performances were observed with rHSP70 (AUC: 0.827, p<0.0001, Likelihood ratio: 8.18) and rH2A (AUC: 0.789, p<0.0001, Likelihood ratio: 2.31), and by SLA (AUC: 0.787, p<0.0001, Likelihood ratio: 7.88). The remaining recombinant proteins (rH2B, rH3, rH4 and rKMP11) presented lower values for AUC and likelihood ratios. When sera from ML patients were evaluated, rHSP70 showed the best performance (AUC: 0.894, p<0.0001, Likelihood ratio: 4.57; Fig. 2B) when compared to the other recombinant proteins (Fig. 2B). With CL sera, rH2A showed the best performance (AUC: 0.808, p<0.0001, Likelihood ratio: 2.52), followed by rHSP70 (AUC: 0.760, p<0.0001, Likelihood ratio: 2.70) and rH2B (AUC: 0.676, p = 0.0004, Likelihood ratio: 2.01) (Fig. 2C).

**Figure 3:**
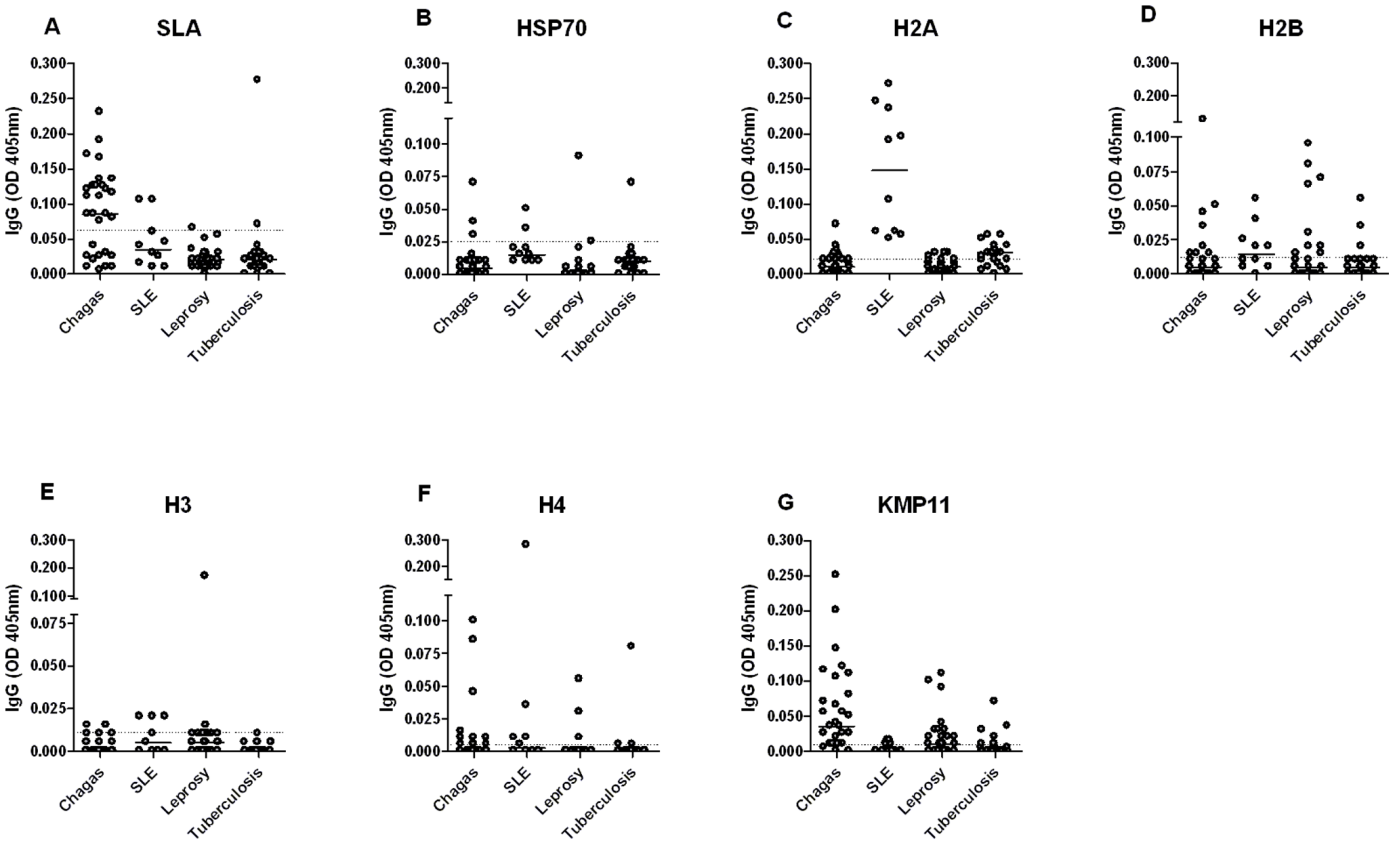
Specificity of ELISA employing parasite antigens. Sera from patients with Chagas' disease (n = 30), SLE (n = 10), leprosy (n = 30) and tuberculosis (n = 22) were tested against SLA (A), rHSP70 (B), rH2A (C), rH2B (D), rH3 (E) rH4 (F) and rKMP11 antigens (G). The cut-off was established from the TL ROC curve (Fig. 2) (dotted line). The solid lines represent median values. Each point represents the mean of duplicate values with a standard deviation lower than 20%.

To evaluate the specificity of tests employing recombinant proteins, serum samples from patients with Chagas disease, leprosy, tuberculosis or SLE were tested against SLA and each recombinant antigen. SLA showed high cross-reactivity against sera from patients with Chagas' disease (63%), but low cross-reactivity for sera from SLE (20%), leprosy (3.3%) or tuberculosis patients (9%) (Fig. 3A). rHSP70 showed the highest reactivity against sera from SLE (20%) or Chagas' disease (10%) patients (Fig. 3B). However, this protein presents lower cross-reactivity values for tuberculosis (4.5%) or leprosy (3.3%) patients sera (Fig. 3B). All the histones tested were highly recognized by sera from SLE patients, especially rH2A which reacted with 100% of tested sera (Fig. 3C–F). Also, the four *Leishmania* recombinant histones showed cross-reactivity with sera from leprosy patients, 23.4% to rH2A (Fig. 3C), 35.7% to rH2B (Fig. 3D), 6.7% to rH3 (Fig. 3E) and 10% to H4 (Fig. 3F). Likewise, for sera from chagasic patients, histones presented similar reactivity: 24.1% for rH2A (Fig. 3C), 26.7% for rH2B (Fig. 3D), 6.7% for rH3 (Fig. 3E) and 23.3% for rH4 (Fig. 3F). Histones also showed low cross-reactivity, (rH2B [13.6% - Fig. 3D] and rH4 [4.5% - Fig. 3F]) or no cross-reactivity (rH3 – Fig. 3E) with sera from tuberculosis patients, except for rH2A where 59% of the samples were recognized (Fig. 3C). For all the antigens tested, rKMP11 antigen showed high cross-reactivity, reaching 86.7%, 40%, 18.2% and 20% with Chagasic, leprosy, tuberculosis and SLE patients' sera, respectively (Fig. 3G). Remarkably, rHSP70 showed the best specificity for all recombinant antigens tested, with similar or lower cross-reactivity when compared with SLA antigen.

**Figure 4:**
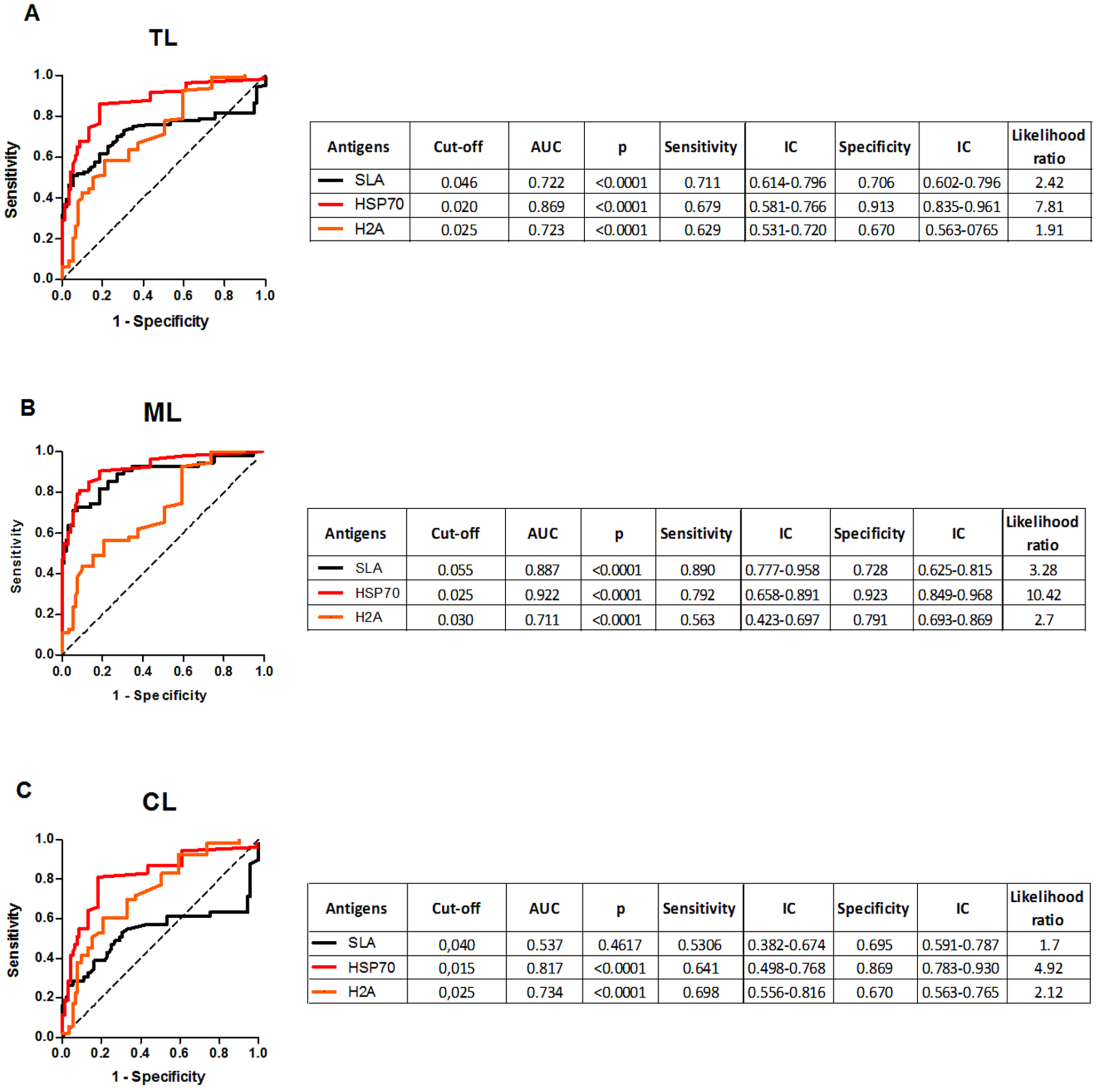
ROC curve of antibody levels predict the positivity thresholds against rHSP70 and rH2A. ROC curves were constructed using data obtained with sera from TL (n = 102) (A), ML (n = 53) (B) and CL (n = 49) as patients and sera from individuals with Chagas' disease (n = 30), SLE (n = 10), leprosy (n = 30) and tuberculosis (n = 22) as negative control. The solid lines represent the area under the curve and the dotted line represents the identity line curves. The tables show the detailed information obtained from each ROC curve (cut-off values chosen, area under the curve, the p values, and the sensitivity and specificity with a confidence interval of 95% and the likelihood ratio).

Based on the previous results, we selected SLA (the gold standard), rH2A (the most reactive histone) and rHSP70 (the best recombinant protein) to perform ROC curves in order to evaluate the performance these parasite antigens in their ability to discriminate patients with Leishmaniasis from those with other pathologies. ROC curves constructed comparing patients with TL and other pathologies showed that the best AUC was obtained with HSP70 (AUC: 0.869, p<0.0001, Likelihood ratio: 7.81) followed by SLA (AUC: 0.722, p<0.0001, Likelihood ratio: 2.42) and rH2A (AUC: 0.723, p<0.0001, Likelihood ratio: 1.91) (Fig. 4A). For sera from ML patients, the best AUC was obtained using rHSP70 (AUC: 0.922, p<0.0001, Likelihood ratio: 10.42) followed by SLA (AUC: 0.887, p<0.0001, Likelihood ratio: 3.28) (Fig. 4B). Also, for sera from CL patients, the highest AUC was obtained with rHSP70 (AUC: 0.817, p<0.0001, Likelihood ratio: 4.92) followed by rH2A (AUC: 0.734, p<0.0001, Likelihood ratio: 2.12) (Fig. 4C). Among the recombinant proteins tested, rHSP70 presented the best performance, as noted by the parameters obtained upon ROC analysis, especially regarding the likelihood ratio presented for this antigen (Fig. 4A–C).

## Discussion

In the last decades, an increasing number of recombinant protein candidates have been proposed to replace the crude *Leishmania* antigen (SLA) for the serodiagnosis of leishmaniasis [24], [31]–[34]. Presently, we decided to test the feasibility of using recombinant antigens derived from *L. infantum-chagasi* for the serodiagnosis of TL. Eventhough these proteins are derived from a *Leishmania* species other than *L. braziliensis*, these proteins belong to conserved families among the various species of *Leishmania* [35], enabling its use for serodiagnosis of TL. In the current study, we compared six *L. infantum-chagasi* recombinant proteins and SLA for the serodiagnosis of TL. We decided to use SLA of *L. infantum chagasi* because all recombinant proteins were derived from this species, and it would not be appropriate to compare with SLA of another species. Antigens derived from unrelated parasite species for diagnosis have been used previously for Leishmaniasis diagnosis [17], [36] Several of these proteins have been previously described as promising antigens for the serodiagnosis to VL but were yet to assayed against a panel of sera from TL patients.

In general, CL patients had significantly lower anti-*Leishmania* antibody titers when compared to sera from ML patients. Higher antibody response of ML as compared to CL patients has been reported and may be due to the prolonged course of disease observed in ML [37], [38]. All recombinant proteins were recognized by sera from TL patients (albeit with different OD) and, among them, rHSP70 presented the best performance in terms of sensitivity and specificity, being superior to SLA in the diagnosis of CL. The antigenicity of heat shock proteins in general and that of HSP70 in particular is not surprising in TL patients, since antibodies against the parasite recombinant HSPs have been found in sera of patients with different parasitic diseases [39]–[41]. In addition, other authors have described that rHSP70 is a preeminent antigen present in *Leishmania* [42]–[46]. Specific antibodies against *L. braziliensis* rHSP70 or rHSP83 were found in 95% of sera from ML or CL patients [47]. Similarly, a high recognition of rHSP70 by sera of CL [48] or ML patients has been described [48], [49]. However, both studies indicated that sera from Chagas' disease patients also gave positive responses albeit with lower OD values than sera from Leishmaniasis patients. We can suggest that sensitivity of the ELISA test may explain the lack of cross reactivity between sera from Chagas' disease patients and rHSP70 in the current report. Presently, the cut off value increased the accuracy of the ELISA assay, possibly explaining the lower cross reactivity observed. Lastly, *Leishmania* rHSP70 has 73% of sequence identity with its human orthologue [50]. Inspite of this high degree of sequence conservation, anti-HSP70 antibodies elicited during *Leishmania* infection specifically recognize the parasite rHSP70, without cross-reactivity with the host's HSP70 [47], [51].

We have also tested nucleosomal histones which have been extensively employed in ELISA against sera from human and canine VL. In such studies, histones presented a high specificity and sensitivity and were suggested as useful antigens for VL serodiagnosis [31], [52]–[55]. The use of histones in the diagnosis of CL or ML is less studied. The presence of antibodies against *L. peruviana* rH2B [25] as well as against *L. braziliensis* H1 [56] has been detected in sera from CL patients. In the present study, we show that the recombinant histones are recognized by a moderate percentage of sera from ML or CL patients but also presented moderate cross reactivity with sera from patients from diseases other than Leishmaniasis, mainly by sera from SLE patients. Among the four *L. infantum-chagasi* recombinant histones tested, rH2A presented better recognition.

In the present report, *L. infantum-chagasi* rKMP11 presented a high performance mainly in ML diagnosis, similar to reports with sera from canine or human VL [46], [57]–[59]. Similar to what had been previously reported, there was a high reactivity of rKMP-11 with sera from Chagasic patients [60], which limits the role of KMP-11 in the diagnosis of TL.

Cross-reaction between *Leishmania* and others diseases caused by common antigenic determinants hinders specific TL diagnosis [61], especially in regions where several parasitic diseases are endemic. Taken together, the data reported here show that *L.infantum-chagasi* rHSP70 should be taken into account for the serodiagnosis of American TL using recombinant antigens. rHSP70 and rH2A proteins presented sensitivity similar to SLA (higher in the case of rHSP70). Of note, we have compared the performance of a combination of rHSP70 plus rH2A in an attempt to increase sensitivity against sera from CL patients. Serological analysis and ROC curves using the combined proteins resented a negligible increase compared to the use of rHSP70 alone. Furthermore, the ROC curve parameters were lower for sera from ML patients. On the other hand, the remaining recombinant antigens (rKMP-11, rH2B, rH3 and rH4) did not satisfactorily replace SLA due to the lower sensitivity and/or specificity. Therefore, rHSP70 or, possibly, combinations with different proteins deserve further evaluation in other endemic areas as a valuable diagnostic tool for the serodiagnosis of TL.
